# Combined H5ND inactivated vaccine protects chickens against challenge by different clades of highly pathogenic avian influenza viruses subtype H5 and virulent Newcastle disease virus

**DOI:** 10.14202/vetworld.2019.97-105

**Published:** 2019-01-01

**Authors:** Ahmed Ali, Marwa Safwat, Walid H. Kilany, Abdou Nagy, Awad A. Shehata, Mohamed A. Zain El-Abideen, Al-Hussien M. Dahshan, Abdel-Satar A. Arafa

**Affiliations:** 1 Department of Poultry Diseases, Faculty of Veterinary Medicine, Beni-Suef University, Beni-Suef 62511, Egypt; 2 Reference Laboratory for Veterinary Quality Control on Poultry Production, Animal Health Research Institute, Dokki, Giza 12618, Egypt; 3 Department of Virology, Faculty of Veterinary Medicine, Zagazig University, Zagazig 44511, Egypt; 4 Department of Veterinary Medicine, Virginia-Maryland Regional College of Veterinary Medicine, University of Maryland, College Park, MD, 20742, USA; 5 Department of Avian and Rabbit Diseases, Faculty of Veterinary Medicine, University of Sadat City, Menoufia 22857, Egypt

**Keywords:** avian influenza, Egypt, H5N1, H5N8, H5ND, Newcastle diseases virus, trivalent vaccine

## Abstract

**Aim:**

The aim of the current study was to evaluate the efficacy of a trivalent-inactivated oil-emulsion vaccine against challenge by different clades highly pathogenic avian influenza (HPAI) viruses including HPAI-H5N8 and the virulent genotype VII Newcastle disease virus (NDV) (vNDV).

**Materials and Methods:**

The vaccine studied herein is composed of reassortant AI viruses rgA/Chicken/Egypt/ME1010/2016 (clade 2.2.1.1), H5N1 rgA/Chicken/Egypt/RG-173CAL/2017 (clade 2.2.1.2), and “NDV” (LaSota NDV/CK/Egypt/11478AF/11); all used at a concentration of 10^8^ EID_50_/bird and mixed with Montanide-ISA70 oil adjuvant. Two-week-old specific pathogen free (SPF) chickens were immunized subcutaneously with 0.5 ml of the vaccine, and hemagglutination inhibition (HI) antibody titers were monitored weekly. The intranasal challenge was conducted 4 weeks post-vaccination (PV) using 10^6^ EID_50_/0.1 ml of the different virulent HPAI-H5N1 viruses representing clades 2.2.1, 2.2.1.1, 2.2.1.2, 2.3.4.4b-H5N8, and the vNDV.

**Results:**

The vaccine induced HI antibody titers of >6log_2_ against both H5N1 and NDV viruses at 2 weeks PV. Clinical protection against all HPAI H5N1 viruses and vNDV was 100%, except for HPAI H5N1 clade-2.2.1 and HPAI H5N8 clade-2.3.4.4b viruses that showed 93.3% protection. Challenged SPF chickens showed significant decreases in the virus shedding titers up to <3log_10_ compared to challenge control chickens. No virus shedding was detected 6 “days post-challenge” in all vaccinated challenged groups.

**Conclusion:**

Our results indicate that the trivalent H5ND vaccine provides significant clinical protection against different clades of the HPAI viruses including the newly emerging H5N8 HPAI virus. Availability of such potent multivalent oil-emulsion vaccine offers an effective tool against HPAI control in endemic countries and promises simpler vaccination programs.

## Introduction

Avian influenza (AI) viruses belong to the family Orthomyxoviridae, genus *Influenza* *virus*. To date, 18 hemagglutinin (HA) and 11 neuraminidase subtypes have been reported [1]. During the past decade, poultry industry in Egypt was challenged by exposure to different AI virus subtypes, including the highly pathogenic AI (HPAI) H5N1, low pathogenic AI H9N2, and HPAI H5N8 [2-4]. Since AI infections emerged in mid-February 2006, HPAI H5N1 showed several mutations and different sub-clades of the virus. The presence of the virus under vaccine immune pressure in vaccinated birds accelerated its mutation rate [5]. Thus, a repertoire of AI virus clades was reported in Egypt since 2008, including 2.2.1, 2.2.1.1, 2.2.1.2, and 2.2.1.2a clades [6-8].

In the meantime, Newcastle disease (ND) continues to cause serious problems and high economic losses in poultry in Egypt. ND virus (NDV) is an avian paramyxovirus serotype 1 belonging to the genus *Avulavirus*, subfamily Paramyxovirinae, family Paramyxoviridae [9]. In Egypt, NDV has been reported since 1948 [10] then the country became endemic. Despite adopting vaccination programs that include both live attenuated and inactivated vaccines, the NDV continues to impact the Egyptian poultry industry [11,12]. The NDV outbreaks are commonly associated with the virulent NDV Genotype VII (vNDV); however, the continuous outbreaks of vNDV were also attributed to poor flock immunity and improper vaccination practices [13,14].

Though over 24 commercial inactivated AI H5 vaccines are licensed for use in poultry in Egypt, the genetic mismatch with poor reactivity of these vaccines to the currently circulating viruses has led to the failure of the HPAI vaccination strategy among poultry in Egypt [3,15]. The spread and co-circulation of different HPAI-H5N1, HPAI-H5N8, and vNDV viruses further complicated the epidemiological situation and control strategies in Egypt with increased economic losses in poultry production. Hence, combined vaccines with matching strains were suggested to facilitate the vaccination programs and minimize the economic losses.

In this study, a trivalent HPAI-H5N1 and NDV-inactivated oil-emulsion vaccine was developed, and its efficacy was evaluated in specific pathogen-free (SPF) broiler chickens against challenge with different clades HPAI-H5N1, HPAI-H5N8 clade 2.3.4.4b, and vNDV virus.

## Materials and Methods

### Ethical approval

Experimental procedures were reviewed and approved by the Animal Care and Use Committee (#171101E001) of the Middle East for Veterinary Vaccines (ME VAC) Company, Egypt.

### Viruses

The vaccine viruses used in this study include the reassortant AI-H5N1 viruses, rgA/Chicken/Egypt/ME1010/2016 (H5N1) “clade 2.2.1.1” (Genbank accession No. MH558951) and rgA/Chicken/Egypt/RG-173CAL/2017 “clade 2.2.1.2” (Genbank accession No. MG192005). Both viruses were developed using reverse genetics system [16] at the Reference Laboratory for Veterinary Quality Control on Poultry Production (RLQP), Animal Health Research Institute, Dokki, Giza, Egypt. Avirulent LaSota-like NDV strain, CK/Egypt/11478AF/2011 (Genbank accession No. MH559344) that was previously isolated and characterized was included in the vaccine [12].

Challenge viruses were selected to represent the different circulating HPAI-H5 virus clades in Egypt, including clade 2.2.1 (A/duck/EG/M2583D/2010, Genbank accession No. CY099580), clade 2.2.1.1 (A/chicken/EG/1063/2010, Genbank accession No. KR732550), clade 2.2.1.2 (A/chicken/EG/1575S/2015, EPI_ISL_174424), and clade 2.3.4.4b (A/common-coot/EG/CA285/2016/H5N8, EPI_ISL_239802). These viruses were designated HPAI H5-2.2.1, HPAI H5-2.2.1.1, HPAI H5-2.2.1.2, and HPAI H5N8-2.3.4.4b, respectively. The vNDV challenge virus NDV/CK/Egypt/567F/2012 (Genbank accession No. JX647839) belongs to genotype VIId currently circulating in Egypt. All viruses were propagated and titrated in 10-day-old SPF eggs.

Both vaccine and challenge HPAI H5 subtype viruses were subjected to phylogenetic and sequence analyses. Phylogenetic relationships were determined with the MEGA version 6 program using the ClustalW alignment algorithm through a bootstrap of 1000 trials [17]. Nucleotide and amino acid sequence analyses were conducted using Geneious® 7.1.3 (Biomatters Ltd., New Zealand).

### Vaccine formulation and testing

The vaccine seed viruses were propagated through inoculation of SPF embryonated chicken eggs through allantoic sac route inoculation. Inoculated eggs were incubated at 37°C for 72 h. Harvested allantoic fluids were clarified with a low-speed centrifuger at 2000 rpm for 10 min at 4°C. The viruses were titrated in 10-day SPF embryonated chicken eggs, and then, hemagglutination (HA) titers and the egg infective dose 50 (EID_50_) were calculated [18,19]. The viruses were inactivated using 0.2% formalin (Sigma-Aldrich, Inc., Germany) and the inactivation was verified by passaging the inactivated antigens into 10-day-old SPF embryonated chicken eggs for three successive passages. The aqueous phase of the vaccine was formulated to contain doses of 10^8^ EID_50_/dose from each virus strain and then mixed with Montanide ISA 70 VG adjuvant (SEPPIC® SA, France) at room temperature with a ratio of 70/30 adjuvant/antigen (v/v). Vaccine physicochemical criteria, safety, and sterility were evaluated according to the SEPPIC Montanide ISA 70 VG technical manual and the OIE standards [19].

### Chicken experiments

In all experiments, White Leghorn SPF chickens kept in biosafety level III chicken isolators were used.

### Birds immunization and challenge

A total of 165, two-week-old SPF chickens were divided into 11 groups (15 birds each) and placed in biosafety level-III chicken isolators. SPF chickens in Groups 1-5 received 0.5 ml/bird of the trivalent vaccine subcutaneously. Groups 6-10 served as challenge controls for HPAI H5-2.2.1, HPAI H5-2.2.1.1, HPAI H5-2.2.1.2, HPAI H5N8-2.3.4.4b, and vNDV viruses, respectively. The last group was inoculated with phosphate buffered saline (PBS) as a negative unvaccinated control (Table-1).

**Table-1:** Experimental grouping and challenge viruses.

Groups	Challenge virus	Parameters
Vaccinated (0.5 ml of trivalent H5ND vaccine S/C)	HPAI H5-2.2.1 (A/DU/EG/M2583D/10-H5N1)	Weekly monitoring of antibody titers
	HPAI H5-2.2.1.1 (A/CK/EG/1063/10-H5N1)	
	HPAI H5-2.2.1.2 (A/CK/EG/1575S/15-H5N1)	Clinical signs
	HPAI H5N8-2.3.4.4b (A/common coot/EG/CA285/16-H5N8)	Mortality
		Virus shedding titers at 3, 6, and 10 dpc
	vNDV Genotype VII NDV/CK/EG/567F/12	
			Non-vaccinated	HPAI H5-2.2.1
HPAI H5-2.2.1.1			
HPAI H5-2.2.1.2			
HPAI H5N8-2.3.4.4b			
vNDV Genotype VII			
PBS negative control			

*HPAI=Highly pathogenic avian influenza, EG=Egypt, CK=Chicken, DU=Duck, S/C=Subcutaneous, vNDV=Virulent Newcastle disease virus, dpc: Days post-challenge, PBS=Phosphate buffered saline

Hemagglutination inhibition (HI) antibody titers were monitored weekly by HI test. Sera of the vaccinated SPF chickens were tested using a clade 2.2.1.2 HPAI-H5N1 antigen (A/duck/EG/M2583D/2010) and a clade 2.3.4.4b HPAI-H5N8 antigen (A/common coot/EG/CA285/2016/H5N8) according to the OIE manual [19]. Virus challenge was conducted 4 weeks post-vaccination (PV) intranasally using 10^6^ EID_50_/0.1 ml of the AI-H5 and vNDV challenge viruses separately. The chosen challenge dose was based on the standard dose being used in Egypt to evaluate all HPAI-H5 and NDV vaccines submitted to the Central Laboratory for Evaluation of Veterinary Biologics, Egypt. Challenged chickens were observed daily for 10 days post-challenge (dpc) for virus shedding and the presence of clinical signs, morbidity, and mortality (Table-1).

### Challenge virus shedding detection

Tracheal swabs were collected from all challenged birds in 1 ml of sterile PBS at 3, 6, and 10 dpc to monitor virus shedding titers. Swab samples were vortexed and centrifuged at 2000 rpm for 10 min at 4°C. Supernatants were used for virus titration in 10-day-old SPF embryonated chicken eggs, and EID_50_/ml was calculated [18].

The cloacal samples were collected, but due to the multiple challenges and large data, we presented the data of tracheal swabs only, especially we did not find significant differences in both types of samples.

### Statistical analysis

Differences in the virus shedding titers at 3 dpc among different groups were calculated using one-way ANOVA with Tukey’s post-test was performed using GraphPad Prism version 5.00 (GraphPad Software, San Diego, California, USA).

## Results

### Genetic analysis of the vaccine and challenge viruses

**Figure-1:**
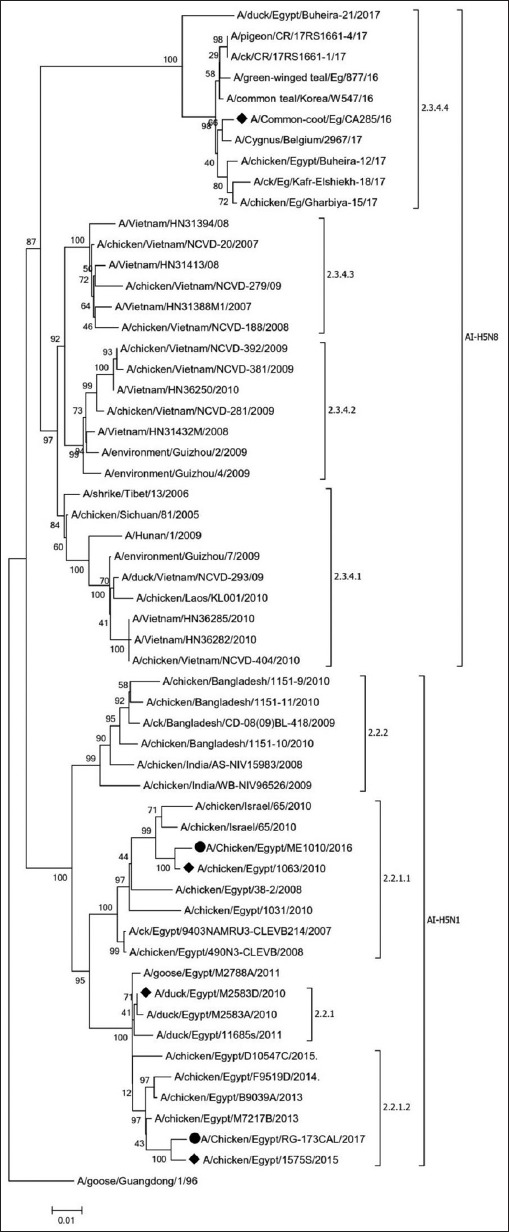
Phylogenetic analyses of hemagglutinin gene sequences of highly pathogenic avian influenza (HPAI) H5N1 vaccine strains (black dots) and HPAI H5N1 and H5N8 challenge strains (black rectangles) compared to the different strains from GenBank using neighbor-joining method with bootstrap values for n=1000 replicates.

Phylogenetic analysis of the HA gene of HPAI H5N1 and HPAI H5N8 viruses included in the current study revealed the clustering of the Egyptian H5N1 viruses into three distinct clades (2.2.1, 2.2.1.1, and 2.2.1.2) and the separate clustering of HPAI H5N8 into clade 2.3.4.4. Selected challenge viruses were confirmed to represent the currently circulating HPAI H5 subtype AI viruses (Figure-1). Nucleotide and amino acid sequence analyses of the vaccine seed strains and HPAI H5N8-2.3.4.4b revealed 88.7-88.8% and 89.8-91.4% identities, respectively. Compared to HPAI H5N8-2.3.4.4b, the A/CK/EG/ME1010/16 and A/CK/EG/RG-173CAL/17 vaccine viruses shared 10 amino acids at the studied antigenic sites and showed 16 and 18 amino acid differences, respectively (Table-2).

**Table-2:** Comparison of the amino acid residues at previously reported antigenic sites in the vaccine and challenge strains.

Item		Vaccine viruses		Challenge viruses			

		A/CK/EG/ME1010/16 2.2.1.1	A/CK/EG/ 173CAL/17 2.2.1.2	A/DU/EG/M2583D/10 2.2.1	A/CK/EG/ 1063/10 2.2.1.1	A/CK/EG/ 1575S/15 2.2.1.2	A/common coot/EG/CA285/16 2.3.4.4b
**Amino acid identity % to 2.3.4.4b H5N8**		**89.8**	**91.4**	**91.7**	**90.2**	**91.2**	**-**
Antigenic sites*	43	D	N	N	D	N	D
	71	P	L	L	P	L	I
	115	K	Q	Q	K	Q	L
	117	I	I	I	I	I	I
	119	K	K	K	K	K	K
	120	S	D	D	S	D	S
	123	P	S	S	P	S	P
	124	D	D	D	D	D	N
	126	E	E	E	E	E	E
	127	A	A	A	A	A	T
	129	L	-	-	L	-	L
	138	Q	Q	Q	Q	Q	Q
	140	G	R	R	G	R	T
	141	P	S	S	S	S	P
	144	Y	F	F	Y	F	F
	151	I	T	T	I	T	I
	154	N	N	N	N	N	N
	155	N	G	D	N	D	D
	156	T	A	A	T	A	A
	158	P	P	P	P	P	P
	159	T	T	T	T	T	T
	162	E	K	K	E	K	I
	163	S	S	S	S	S	S
	165	H	N	N	H	N	N
	174	V	V	V	V	V	I
	181	P	P	P	P	P	S
	189	R	R	R	R	R	N
	190	I	L	L	I	L	L
	192	K	Q	Q	K	Q	K
	195	T	T	T	T	T	T
	198	I	I	I	I	I	I
	223	S	S	S	S	S	R
	226	V	M	M	V	M	M

* Residues in the vaccine strains that are identical to HPAI H5N8-2.3.4.4b (A/common coot/EG/CA285/2016) are gray shaded, the studied antigenic sites are previously reported by Duvvuri *et al*., Kaverin *et al*., Kaverin *et al*., Khurana *et al*. and Sun *et al*. [33-37]. HPAI=Highly pathogenic avian influenza, EG=Egypt, CK=Chicken, DU=Duck, PBS=Phosphate buffered saline

### Humoral immune response in vaccinated chickens

Vaccinated SPF chickens elicited detectable HI antibody titers for HPAI-H5N1 and vNDV by 2 weeks PV using HPAI-H5N1 and NDV antigens (log2 6.0±1.1 and 6.6±0.9, respectively). The HI antibody titers using the heterologous HPAI-H5N8-2.3.4.4b were observed 3 weeks PV in 7 out of 15 birds, but all were seropositive 4 weeks PV (titers of 5.0±1.0 log2, Table-3).

**Table-3:** Mean HI antibody titers and seropositivity percentages in vaccinated birds.

Weeks PV	Type of Antigen used for HI test^1^					

	HPAI H5N1-2.2.1		HPAI H5N8-2.3.4.4b		NDV-Ag	
		
	Mean±SD^2^	Positivity^3^%	Mean±SD	Positivity %	Mean±SD	Positivity %
1	0.0±0.0	0	0.0±0.0	0	0.0±0.0	0
2	6.0±1.1	100	0.0±0.0	0	6.6±0.9	100
3	9.2±0.8	100	2.6±0.8	46.7	8.4±0.6	100
4	9.8±0.5	100	5.0±1.0	100	8.9±0.6	100

1 HI=Hemagglutination inhibition test, HPAI H5-2.2.1 AI-H5N1 antigen=A/duck/EG/M2583D/2010, HPAI H5N8-2.3.4.4b antigen (A/common coot/EG/CA285/2016/H5N8). HPAI=Highly pathogenic avian influenza,

2 SD=Standard deviation,

3 Positivity %=Number of seropositive birds/total tested×100. PV=Post-vaccination, HI=Hemagglutination inhibition

### Protective vaccine efficacy against different challenge viruses

#### Clinical protection

**Figure-2:**
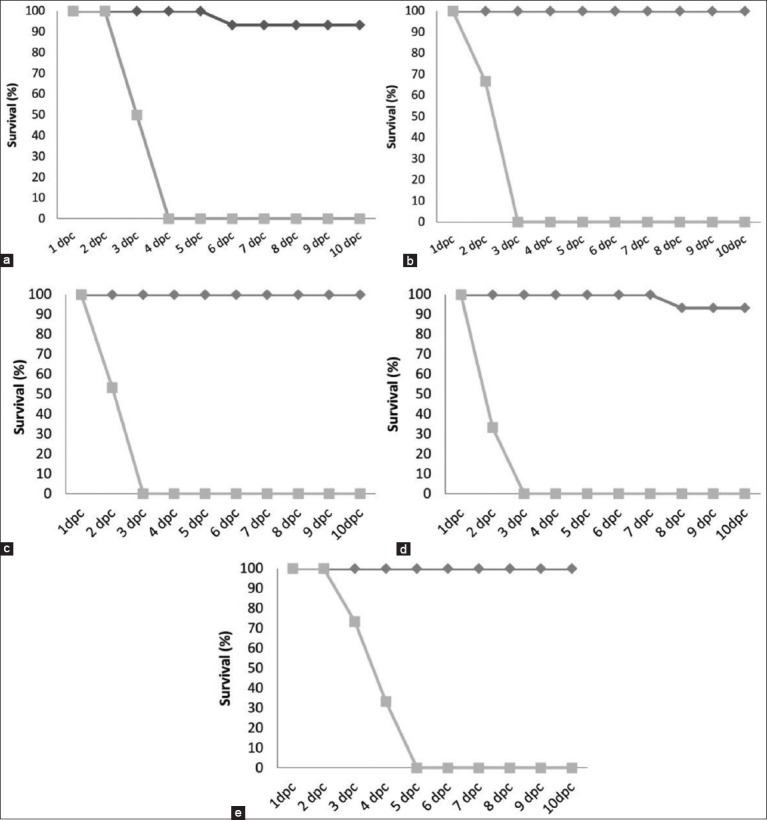
(a-e) Survival rates in the H5ND-vaccinated birds post-challenge with different clades highly pathogenic avian influenza H5N1, H5N8, and virulent Newcastle disease viruses.

The negative control group did not show any clinical signs during the experiment. The HPAI-H5 challenge control birds demonstrated the typical clinical signs and post-mortem lesions by 2 dpc including edema and cyanosis of the comb and wattles and hemorrhage on shanks. Mortality reached 100% by 3 dpc. Typical vNDV clinical signs, including severe respiratory manifestations and mortalities, observed at 3 dpc with 100% mortalities by 5 dpc. In vaccinated groups, the protection levels were 100% against HPAI H5-2.2.1.1, HPAI H5-2.2.1.2, and vNDV challenge viruses (Figure-2b, c, and e); whereas, protection levels against HPAI H5-2.2.1 and HPAI H5N8-2.3.4.4b challenge were 93.3% (Figure-2a and d). Notably, deaths in HPAI H5N8-2.3.4.4b control challenge group were observed as early as 2 dpc, and all chickens died by 3 dpc (Table-4).

**Table-4:** Challenge virus shedding titers in vaccinated and non-vaccinated challenged control groups.

Group	dpc^1^	Challenge virus shedding titers EID_50_/ml (number of positives/total tested)^2^				

		HPAI H5-2.2.1	HPAI H5-2.2.1.1	HPAI H5-2.2.1.2	HPAI H5N8-2.3.4.4b	vNDV
H5ND- vaccinated groups	3	2.1±0.66^a^ (5/15)	2.2±0.76^a^ (3/15)	1.8±0.81^a^ (4/15)	1.3±0.01^a^ (3/15)	2.1±0.3^a^ (6/15)
	6	2.8±0.0 (1/14)	0 (0/15)	0 (0/15)	1.7±0.0 (1/15)	1.9±0.5 (2/15)
	10	0 (0/14)	0 (0/15)	0 (0/15)	0 (0/14)	0 (0/15)
Challenge control^4^	2	NT^3^	NT	NT	4.5±0.6	NT
	3	5.8±0.47^b^ (15/15)	5.7±0.72^b^ (15/15)	5.9±0.54^b^ (15/15)	3.9±0.4^b^ (5/5)	4.8±0.7^b^ (15/15)
	4	5.7±0.46 (4/4)	--	6.4±0.31 (5/5)	--	4.3±0.6
	5	--	--	--	--	5.1±0.7
	6	--	--	--	--	--
	10	--	--	--	--	--
PBS negative control	All	Not detected				

1 dpc=Days post-challenge-additional days in challenge control groups are the days at which deaths of infected birds occur;

2 Virus shedding titers at the same column at ^3^dpc and followed by different superscript small letters indicate significant differences (p≤0.05);

3 NT=Not tested,

4 All birds died by 3–4 dpc, HPAI=Highly pathogenic avian influenza, PBS=Phosphate buffered saline

### Challenge virus shedding

In all vaccinated groups, the reduction of virus shedding titers was significant at 3 dpc (≥3.5 log_10_ EID_50_/ml, p≤0.05) with a very low number of shedding birds compared to the corresponding HPAI H5 clades challenge control groups. Although 33% of chickens challenged with HPAI H5-2.2.1 shed the virus with titers of 2.1±0.6 log_10_ EID_50_/ml at 3 dpc, this percentage was reduced by 6 dpc to 7.1% (1 out of 14 birds). Similarly, 20% of vaccinated chickens challenged with HPAI H5N8-2.3.4.4b shed the virus at 3 dpc, then the percentage decreased to 6.7% (1 out of 15 birds) by 6 dpc (1.7 log_10_ EID_50_/ml). The other two vaccinated groups challenged with HPAI H5-2.2.1.1 and 2.2.1.2 viruses showed no virus shedding by 6 dpc. Similarly, the vNDV challenge group exhibited significantly reduced virus shedding titers that further diminished by 6 dpc (Table-4).

## Discussion

Inadequate biosecurity and relying on vaccination as the only control strategy for the HPAI-H5N1 virus in Egypt led to frequent mutations of the virus and evolution of different subclades, especially with the use of mismatch vaccine strains [3,20]. In late 2016, the epidemiology of AI in Egypt exhibited a substantial change due to the emergence of HP H5N8 in wild birds [4] followed by widespread of the virus in commercial poultry [21,22]. Moreover, other poultry pathogens including vNDV and infectious bronchitis virus became more frequently diagnosed in poultry in Egypt [23].

An immunization strategy depending on using bivalent and multivalent vaccines containing whole inactivated viruses has been advocated before to control several avian pathogens [24]. The objective of the current study was to evaluate the immunogenicity and protective efficacy of a trivalent-inactivated oil-emulsion H5ND vaccine against different clades HPAI subtype H5 and vNDV viruses following a single-dose vaccinationregimen. The vaccine contained two reassortant H5N1 viruses representing both 2.2.1.1 and 2.2.1.2 clades registered in Egypt and a LaSota-like NDV strain.

In the vaccination challenge experiments, the vaccine-induced HI antibody titers by 2 weeks post-vaccination against both HPAI-H5N1 and NDV antigens. However, the HI antibody titers against HPAI H5N8 heterologous antigens were only detectable at very low titers 3 weeks PV. By 4 weeks PV, the anti-HPAI H5N8 antibody titers were ≥5.0 log2. These relatively low antibody titers were rather expected due to the genetic and antigenic differences in the HA between HPAI H5N1 and H5N8 viruses isolated in Egypt [25,26].

The protective efficacy of the developed H5ND vaccine was evaluated by challenge of vaccinated birds with 10^6^ EID_50_/100 µl of the different clades HPAI AI viruses or vNDV at 4 weeks post-vaccination (PV). All non-vaccinated chickens showed severe clinical signs and 100% mortality by 3 and 4 dpc in all HPAI and vNDV virus challenge groups, respectively. Although few reports indicated that HPAI H5N8 viruses produce asymptomatic disease in geese and ducks with prolonged virus shedding [27], increased virus adaptation to chickens was observed within the HPAI of 2.3.4.4 clade viruses [28,29]. This was supported by the finding that members of the HPAI H5N8 challenge group showed typical AI signs and 100% mortality rate.

In terms of clinical protection, 100% of the vaccinated chicken groups survived challenge with HPAI H5-2.2.1.1, HPAI H5-2.2.1.2, and vNDV. Protection against HPAI H5-2.2.1 was 93.3% accompanied by reduced shedding titers after challenge. Previous reports of an antigenic distinction between subclades 2.2.1 and 2.2.1.1 associated with substantial antigenic drifts [30,31] may explain the 6.7% mortality observed.

There was a significant (p<0.01) reduction in both virus shedding titers and the number of active virus shedders in all challenged groups, including those receiving HPAI H5-2.3.4.4b, despite the low HI antibody titers against HPAI-H5N8-2.3.4.4 clade virus. In contrast, Yuk *et al*. [32] showed that while commercial clade 2.3.2 H5 vaccines protected chickens against HPAI-H5N8 virus challenge, they failed to prevent virus shedding. It is worthwhile to note that the seed virus of clade 2.3.2 viruses showed 84.6-87.7% amino acid identities with the HPAI H5N8 challenge virus, compared to 89.8-91.4% in the current study.

In another study, the efficacy of commercial vaccines available in Egypt was studied. Most of the vaccines did not exceed 80% of protection and did not prevent virus shedding with the exception of a commercial vaccine based on a clade 2.3.4 H5N1 virus that reduced virus shedding [25]. In this study, we used two HPAI-H5N1 viruses belonging to two different clades that are 89.8-91.4% identical to the HPAI H5N8-2.3.4.4 on the amino acid level. In addition, both viruses share several amino acids that are distributed at the previously reported antigenic sites [33-37]. The aforementioned factors may explain the observed protection, relatively better HI antibody titers, and reduced virus shedding titers, compared to previous studies [25,38,39]. Moreover, previous reports indicated that changes in HA amino acids may not correspond to both clade and subclade grouping and the protective efficacy of vaccine preparations [35].

Although the protection rate (≥90%) against HPAI-H5N8-2.3.4.4b is acceptable for a combined vaccine and the vaccine could be regarded as effective, this finding cannot be extrapolated to the field conditions considering the first 3 weeks gap observed until a relatively high cross antibody titers are detected. The complicated poultry field situation and inadequate biosecurity measures may reduce the efficacy of the HPAI H5N1 vaccines against HPAI-H5N8-2.3.4.4b compared to experimental studies. Therefore, studies of different vaccination strategies (e.g., double dose regimen) and/or vaccine development for better control of clade 2.3.4.4b HPAI H5N8 in Egypt may be needed [25].

## Conclusion

The trivalent H5ND vaccine was found to be immunogenic, and it provides protection in SPF chickens against HPAI H5 AI and virulent ND infections. The current study also demonstrates that the multivalent oil-emulsion vaccines could be a useful strategy to simplify the vaccination programs for controlling multiple poultry viruses, especially in endemic countries.

## Authors’ Contributions

AA, MS, WHK, AN, and AAA: Conceptualization and design of the study. AA, MS, WHK, AN, AAS, MAZ, and AMD: Conducted the experiment, analyzed, and interpreted the data. AA, WHK, and AAA: Writing and revising the manuscript. All authors: Reviewing, editing, and approved the final manuscript.
